# Dimethylsulfoniopropionate (DMSP) Increases Survival of Larval Sablefish, *Anoplopoma fimbria*

**DOI:** 10.1007/s10886-016-0713-z

**Published:** 2016-06-15

**Authors:** Jonathan S. F. Lee, Rachel S. Poretsky, Matthew A. Cook, Jose J. Reyes-Tomassini, Barry A. Berejikian, Frederick W. Goetz

**Affiliations:** Environmental and Fisheries Sciences Division, Northwest Fisheries Science Center, National Marine Fisheries Service, NOAA, 7305 Beach Dr E, Port Orchard, WA 98366 USA; Department of Biological Sciences, University of Illinois at Chicago, 845 W. Taylor Street, Chicago, IL 60607 USA

**Keywords:** Feeding stimulant, Larvae, Fish, Phytoplankton, Recruitment

## Abstract

High concentrations of dimethylsulfoniopropionate (DMSP), a chemical compound released by lysed phytoplankton, may indicate high rates of grazing by zooplankton and may thus be a foraging cue for planktivorous fishes. Previous studies have shown that some planktivorous fishes and birds aggregate or alter locomotory behavior in response to this chemical cue, which is likely adaptive because it helps them locate prey. These behavioral responses have been demonstrated in juveniles and adults, but no studies have tested for effects on larval fish. Larvae suffer from high mortality rates and are vulnerable to starvation. While larvae are generally thought to be visual predators, they actually have poor vision and cryptic prey. Thus, larval fish should benefit from a chemical cue that provides information on prey abundance. We reared larval sablefish, *Anoplopoma fimbria*, for one week and supplemented feedings with varying concentrations of DMSP to test the hypothesis that DMSP affects larval survival. Ecologically relevant DMSP concentrations increased larval survival by up to 70 %, which has implications for production in aquaculture and recruitment in nature. These results provide a new tool for increasing larval production in aquaculture and also suggest that larvae may use DMSP as an olfactory cue. The release of DMSP may be a previously unappreciated mechanism through which phytoplankton affect larval survival and recruitment.

## Introduction

Dimethylsulfoniopropionate (DMSP) and dimethylsulphide (DMS) are chemical cues for some planktivorous fishes and birds (DeBose et al. 2008; Nevitt 2011). Since DMSP is released from phytoplankton upon grazing by zooplankton and converted to DMS by bacteria, high concentrations of these chemicals can indicate high concentrations of planktonic prey. Some planktivores aggregate or change rates of locomotion in response to ecologically relevant concentrations of DMSP (up to 100 nM, DeBose et al. 2008). Further, extremely high DMSP concentrations (10^−5^ to 10^−2^ M) can increase feeding strikes and growth in captivity (Nakajima et al. 1990). By providing information about prey densities, these chemical cues may allow planktivores to increase energetically expensive foraging and feeding behaviors adaptively, when those energetic costs can be offset by increased acquisition of prey. The ability to respond to DMSP is taxonomically widespread, but less attention has been paid to the diversity of developmental stages that respond. Specifically, no study has tested whether DMSP affects larval fish.

Dimethylsulfoniopropionate may have important implications for larvae in both nature and aquaculture. Larval fish are thought to be visual predators, but have an extremely limited visual range and cryptic prey (Utne-Palm 2002). Thus, olfactory detection of DMSP could potentially be a major modality through which larvae detect prey. In nature, where larvae are especially vulnerable to death from starvation, DMSP may improve larval survival and recruitment. The compound also could be important for marine aquaculture, where the larval stage is a major bottleneck. Larval feeding transitions are especially difficult, particularly during early larval life when larvae deplete their yolk sacs and must learn to feed, and during weaning periods between food types. This study tested whether first-feeding larvae of sablefish, *Anoplopoma fimbria*, which is a candidate species for aquaculture, survive at higher rates in the presence of DMSP.

## Methods and Materials

Each of 24 tanks (36.5 cm diam x 43.0 cm tall, 37.0 L) was stocked with first-feeding larvae that originated from a broodstock cross of sablefish collected from the Washington coast. For collection, spawning, and early rearing details, see Cook et al. (2015). Tanks had black side walls and white bottoms. Filtered seawater (12 °C) flowed into each tank at 270 ml/min and out through a center standpipe. Tanks were enveloped by a 38.7-cm diam cylinder made of reflective aluminum-lined bubble wrap that extended 58.4 cm above each tank. A white plastic disk rested on top of each cylinder and housed a 3.8 watt LED light bulb (15 lx at the water surface, Luxxo MR16, 3.8 60° CW, Kumho Electric, Santa Fe Springs, CA, USA). Throughout this experiment, peristaltic pumps delivered concentrated solutions of claywater (total tank concentration: 12 mg clay per L seawater, Kentucky Ball Clay OM4) to each tank to generate turbidity and improve the visual environment. Like larvae of many marine species (Utne-Palm 2002), sablefish larvae have difficulty seeing without turbidity.

First-feeding larvae were stocked volumetrically—a 19 L bucket was filled with larvae and filtered seawater from the source tank, then the density of larvae was estimated from a 200 ml sample. The appropriate water volume was transferred from the bucket to each experimental tank to achieve approximately 520 larvae per tank. Within an hour after stocking and before DMSP treatments had begun, a visual inspection indicated some mortality that appeared uneven among replicate tanks. Because of the imprecise volumetric stocking method and the immediate, uneven mortality, photographs were taken of each tank surface (camera pointing down from above) for surface stocking counts. Previous experience has shown that surface stocking counts (# larvae visible from surface) correlate with the number of larvae in the tank, so surface stocking counts allow later survival data to be corrected for actual initial stocking numbers. After the initial mortality, the surviving larvae appeared normal—swimming and feeding in the same manner as in previous experiments we have conducted over the past several years. There also was an algae treatment (algae plus seawater and no added DMSP), but data from that treatment were excluded because surface stocking counts are not comparable between water with algae vs. clay.

Dimethylsulfoniopropionate was provided by Prof W.B. Whitman, Laboratory of Microbiology, University of Georgia. Rotifers, *Branchionus plicatilis*, were supplied as food twice per day (25 rotifers/ml of tank water). Fifteen and 135 min after each feeding, either high-concentration DMSP (DMSP-HIGH, 100 nM tank concentration; *N* = 8), low-concentration DMSP (DMSP-LOW, 1 nM tank concentration; *N* = 8), or water (WATER; *N* = 8,) was added to each tank. On the eighth day, all tanks were drained and surviving larvae were counted. To standardize survival to surface stocking counts, we divided the number of survivors in each tank by the surface stocking counts taken at the beginning of the study (“relative survival rate”). An ANOVA tested for differences in surface stocking counts among treatments (α = 0.05). Another ANOVA followed by Tukey-Kramer HSD tested for differences in relative survival rate among treatments (α = 0.05). All data met assumptions for normality and homogeneity of variances.

In order to get a sense of DMSP dynamics in the tanks, a single dose of DMSP-HIGH, DMSP-LOW, or WATER was added to study tanks under conditions identical to the rearing study, except no larvae were present (*N* = 3 tanks per treatment). Water samples were taken 10 min after water addition in the WATER treatment, and 10 min, 2 h, and 4 h after DMSP addition for DMSP-HIGH and DMSP-LOW treatments. DMSP was quantified using the procedure described by Kiene and Slezak (2006). Briefly, after acidifying 10 ml of each sample to a 1.5 % HCl concentration to preserve DMSP, DMSP samples were analyzed on a Shimadzu GC-2014 gas chromatograph equipped with a flame photometric detector and packed column of Chromosil 330 (Sigma; 1/8″ dia Teflon x 2 m length). The column oven was maintained at 60 °C and the carrier gas was helium at 25 ml min^−1^. Injector and detector temperatures were 175 °C. Dimethyl sulfide (DMS) eluted at a retention time of 1.7 min and the minimum detection limit was 1 pmol DMS per injection. Calibration was carried out with a DMS standard generated by a permeation system. Sample volume was adjusted to bring DMS peak areas into the calibrated range.

## Results and Discussion

**Fig. 1:**
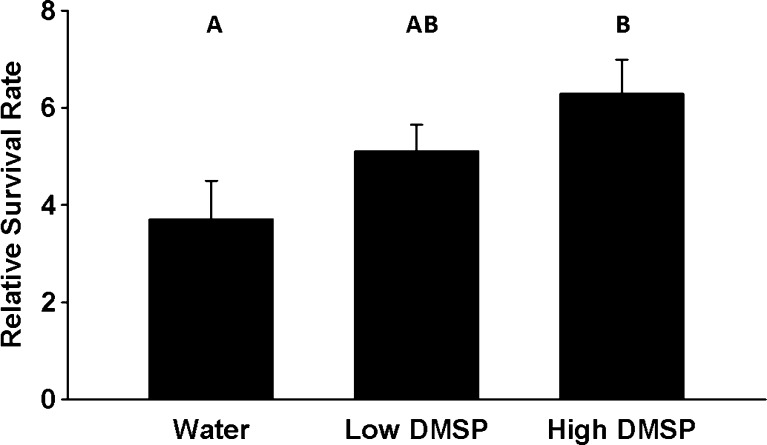
Relative survival rate with varying concentrations of dimethylsulfoniopropionate (DMSP). Error bars represent standard error. Different letters represent significantly different treatments (*P* < 0.05)

Surface stocking counts taken at the beginning of the experiment were not significantly different among treatments (*P* = 0.958; average, range for WATER: 40.1, 17–63; DMSP-LOW: 39.3, 17–74; DMSP-HIGH: 41.6, 23–62). Relative survival rates differed significantly among treatments (ANOVA: *P* = 0.0499, Fig. 1). DMSP-LOW was not significantly different from WATER (*P* = 0.344) or DMSP-HIGH (*P* = 0.465), but DMSP-HIGH had a significantly higher relative survival rate than WATER (*P* = 0.040). DMSP concentrations varied among sampled tanks (Table 1). DMSP in the water treatment indicates that it was present in limited concentrations in the seawater that was pumped in from Puget Sound.

**Table 1:** Dimethylsulfoniopropionate (DMSP) concentrations at different time points in tank water resulting from a single dose of DMSP-HIGH, DMSP-LOW, or WATER. Time points represent elapsed time between treatment dosing and water sampling

	DMSP concentrations (mean in nM, standard error)		
Treatment	10 min	2 h	4 h
Water	2.60 (0.04)	not measured	not measured
DMSP-LOW	3.42 (0.11)	3.26 (0.09)	4.03 (0.09)
DMSP-HIGH	61.60 (2.31)	22.55 (0.87)	11.75 (0.19)

While previous studies have focused on foraging behaviors in juveniles and adults, this study found effects of ecologically-relevant DMSP concentrations on survival. For example, 1–10 nM generally is measured in surface seawater and >100 nM in blooms of DMSP-producing algae. Survival is a metric that is closely related to ultimate measures of success for recruitment in nature and production in aquaculture. Studies in other species suggest that this effect on survival may have been mediated through DMSP effects on feeding behavior (DeBose et al. 2008; Nakajima et al. 1990; Nevitt 2011). Our experimental design excludes the possibility that DMSP served as a chemoattractant, but DMSP may have increased other aspects of larval feeding behavior such as activity or feeding strike frequency. Increased feeding strike frequency has been demonstrated in juvenile red sea bream and yellowtail exposed to DMSP (Nakajima et al. 1990). Dimethylsulfoniopropionate also could have affected rotifer behavior. For example, it could have increased rotifer activity, making them more susceptible to capture by larval sablefish. The survival benefit to the larval sablefish was large (70 % greater survival in the DMSP-HIGH treatment vs. control) and occurred during the early larval period when larvae deplete their egg yolk reserves and must learn to feed on live prey (Rao 2003). Changes in larval mortality rates during this major bottleneck period can have large effects on recruitment in nature and production in aquaculture (Leggett and Deblois 1994; Rao 2003).

Dimethylsulfoniopropionate could potentially improve survival during crucial larval rearing periods that commonly limit aquaculture production. In some species, algae are added to rearing water (creating “greenwater”) primarily for visual purposes. The turbidity is thought to improve the visual environment for fish larvae by scattering light or enhancing contrast (Utne-Palm 2002). However, addition of algae is expensive and can decay, leading to increased bacterial growth and water fouling (Attramadal et al. 2012). Attempts to replace algae with less expensive and inorganic clay have met with mixed results (e.g., Daugherty 2013), particularly during the early larval period (unpublished data, Lee et al.; personal communication, aquaculture industry). In aquaculture, as larvae deplete their yolk sacs, DMSP derived from algae may help stimulate predation on live prey, and thus could be a previously unrecognized benefit of greenwater. Clay and DMSP, perhaps at even higher concentrations than those used in this study, may substitute for algae during early larval rearing. Alternatively, algae could be supplemented with DMSP, potentially to generate better survival than with algae alone. Additionally, DMSP also could be useful during later feed transitions, for example during weaning from live feeds to prepared diets.

Dimethylsulfoniopropionate also may affect survival in nature. Uncovering the determinants of inter-annual variation in recruitment has been a primary focus in fish ecology (Subbey et al. 2014). Food availability has been considered a major factor driving recruitment. Increases in phytoplankton abundance can positively affect recruitment (Platt et al. 2003), possibly because some larvae may consume phytoplankton directly, and because increased phytoplankton abundance can lead to increased abundance of zooplankton, which are then consumed by larvae. Our results suggest that DMSP may help mediate the relationship between phytoplankton abundance, zooplankton abundance, and larval survival and recruitment.

In conclusion, this study suggests that DMSP plays a role in increasing larval survival in an aquaculture setting, and that the effects could potentially extend to the natural environment. Future work should explore further the potential roles and uses of DMSP in recruitment and aquaculture in sablefish as well as other species.
